# Effect of different doses of radiation on morphogical, mechanical and chemical properties of primary and permanent teeth—an in vitro study

**DOI:** 10.1186/s12903-020-01222-3

**Published:** 2020-09-01

**Authors:** Gülsüm Duruk, Burçin Acar, Öztun Temelli

**Affiliations:** 1 Department of Pediatric Dentistry, Faculty of Dentistry, Inonu University, Malatya, Turkey; 2 Department of Radiation Oncology, Faculty of Medicine, Inonu University, Malatya, Turkey

**Keywords:** Primary teeth, Permanent teeth, ICP-OES, Microhardness, Radiation

## Abstract

**Background:**

Radiotherapy, applied to the head and neck region, can cause radiation side effects such as reduction of saliva and radiation caries. The aim of this study was to perform an in vitro assessment of the effects of radiation therapy on the morphological, mechanical, and chemical properties of primary and permanent teeth.

**Methods:**

One hundred four extracted human teeth (52 impacted wisdom teeth, 52 primary molar teeth) were used. The teeth were divided into two parts in the mesiodistal direction. Of the 98 teeth, the vestibular sections were used for the vickers analysis and lingual sections were used for the Inductively Coupled Plasma-Optical Emission Spectrometry (ICP-OES) analysis. The teeth in the experimental group were fixed to wax models. Each model had an equal number of teeth (*n* = 7). The doses were applied to the teeth for 6 weeks; 5 week days and 2Gy daily. After the radiotherapy was conducted weekly, a wax model was taken from radiation reception. Along with the elemental contents (Na, K, Mg, P, and Ca) of the teeth, enamel and dentin microhardness was evaluated, and SEM analyzes were performed on 6 teeth.

**Results:**

Radiation caused a decrease in microhardness of enamel and dentin (*p* < 0.05). In the elemental analysis by ICP-OES, it was observed that there were decreases in all elements after 60Gy compared to the control group (*p* < 0.05). In the experimental groups, amorphous structures were encountered in SEM images.

**Conclusions:**

Radiation has negative effects on the teeth structure and additional studies are needed in this regard. This study indicates that radiotherapy patients are at a higher risk for dental caries.

## Significance of the study

In our study, the effects of radiation therapy on teeth were investigated. It was proven in this study that the radiation therapy has side effects on the morphogical, mechanical and chemical properties of the primary and permanent teeth. This study will shed light on future researches about radiation therapy.

## Introduction

According to the reports by Globacan cancer incidence estimation, a total of 18.1 million new cancer cases were reported in the world in 2018 [1]. Head and neck cancer ranks seventh in the world with an incidence of 13.6%. While head and neck cancers are rare in children, thyroid cancers are found to be common [2].

Head and neck cancers are a heterogeneous tumor group, including the oral cavity, larynx, pharynx, thyroid, lips, nasal cavity, paranasal sinuses, and salivary glands. Their treatment includes surgery, radiotherapy, chemotherapy, and mostly the combination of them [3, 4]. Radiotherapy is widely used as a primary treatment, an adjuvant treatment, or a palliative treatment in the last stages of the disease [5].

In radiation therapy, high-energy radioactive elements produced by X-ray equipment and particle accelerators are used. These elements act by directly stimulating the rupture of DNA strands or by indirectly causing the effect of cellular necrosis in the production of hydrogen peroxide resulting from the physical effect of free radicals and gamma irradiation in water [6].

During the radiation therapy in head and neck cancers, healthy surrounding tissues such as bones, mucosa, teeth, and salivary glands are, unfortunately, not well-preserved.

Radiation caries, which is one of the most threatening complications of radiotherapy, is commonly seen. A systematic review reported the average prevalence of radiation caries as 28.1% and the average number of decayed, missing, and filled teeth (DMFT) in patients after irradiation as 9.19 [7]. Radiation caries leads to severe destruction of mineralized tooth tissues and progresses rapidly, unlike conventional caries lesions [8]. A caries lesion begins in the form of the exposed dentine after an enamel fracture and enamel loss [8]. Dentinoenamel junction (DEJ) plays an important role in the pathological process of radiation caries. Changes in the amount and composition of saliva are among the major causes of radiation caries [9]. However, this does not fully explain the causes of enamel fractures. This obscurity has forced researchers to investigate the direct effects of radiation on dental hard tissues [4, 10–15]. However, the number of studies on how teeth structure changes with radiotherapy is insufficient nowadays, and there is still no consensus in studies [4, 10–13, 15–17].

It is important to study the effect of radiation on teeth. So, more effective strategies will be developed to prevent radiation caries and achieve better results in these patients’ oral health.

The aim of this study was to evaluate the effects of radiation on the mechanical, morphological, and chemical properties of primary and permanent teeth.

The null hypothesis of this study is that (i) there is no statistically significant difference between the noniradiated teeth and the teeth exposed to radiation in terms of elemental content, (ii) there is no statistically significant difference between the noniradiated teeth and the teeth exposed to radiation in terms of microhardness.

## Methods

According to the power analysis, in order to calculate the changes of microhardness and Ca element of the teeth caused by the increase of each 10Gy, the estimated number of samples was 6 per group, with an alpha level of 0.05 and a power of 0.80. The approval of this study was obtained from the ethics committee (2014/82). In our study, impacted permanent third molar teeth having indicated the extraction were used. Primary molar teeth with the indication of the extraction were also used due to the physiological root resorption of the patients who applied to Inönü University, Faculty of Dentistry, Oral and Maxillofacial Surgery Clinic. One hundred four extracted human teeth were stored at + 4 °C in distilled water.

**Inclusion criteria**

- The teeth of the individuals who live in the same geographic area, who did not have systemic or genetic problems, and who had not been exposed to radiation before.
- The teeth which have no enamel/dentin/enamel+dentin caries or no restorative material (filling or fissure sealant).
- The teeth which have no hypomineralized and/or hypocalcified areas, and no an abrasion that will expose the dentin surface.

### Experimental design

Samples were divided into two categories as primary and permanent teeth. In each category, the groups were formed to include 7-tooth samples for each dose of radiation (from 10Gy to 60Gy; six groups) and for control group. Fourty-nine samples (7 groups × 7 samples = 49) were used for ICP-OES analysis. Similarly, 49 samples were used for the Vickers analysis. For SEM, three primary and three permanent teeth for control, 30 and 60Gy radiotherapy (*n* = 6) were used.

### Sample preparation

The teeth samples were prepared by one examiner. Before the study, the examiner was trained and calibrated.

**Fig. 1:**
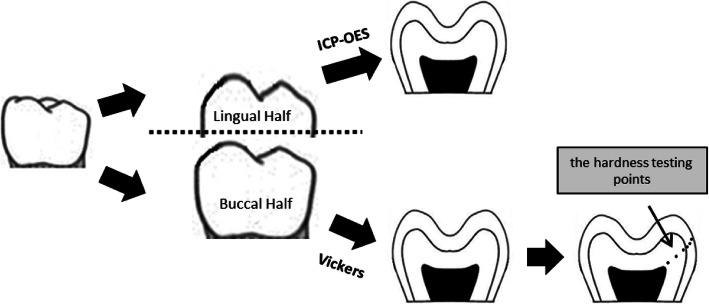
Figure showing the sites where the mechanical property and the chemical structure data were collected in relation to the buccal and lingual half of the tooth

The roots of the teeth were removed with a water-cooled diamond disc, and the crowns were divided into two parts in the mesiodistal direction. The vestibular part was used for the Vickers analysis, and the palatinal part was used for the ICP-OES analysis (Fig. 1).

### Radiation application

The teeth in the experimental group were fixed to wax the patterns (7 teeth fixed to each wax pattern) and covered with a gauze patch impregnated with artificial saliva and then placed in the center of a styrofoam container filled with partially crushed rice. Five different styrofoam containers were prepared (ICP-OES-primary teeth, ICP-OES-permanent teeth, Vickers-primary teeth, Vickers-permanent teeth, SEM). Radiation (6 MV X-ray) was applied to the teeth 5 days (2Gy per day) a week during 6 weeks. This protocol called RTOG 95–01 (60Gy in 6 weeks - 2Gy once a day, 5 x a week) was designed and developed by the Radiation Therapy Oncology Group (RTOG) of the American College of Radiology (ACR) [18]. Radiation therapy was performed with the SAD technique at a dose rate of 400 MU/min with the anterior and posterior areas. At the end of each week, a wax pattern was removed from radiation intake. After each radiotherapy application, the teeth were taken from the container full of rice, and placed in artificial saliva, and then kept in the oven at 37 °C. This procedure was repeated every day when exposed to radiation.

Radiotherapy was applied to the teeth by a technician under the supervision of a radiation oncologist using the linear accelerator (Clinac iX, Varian, CA, USA) in the Radiation Oncology Department of Inönü University Faculty of Medicine.

### Inductively coupled plasma optic emission spectroscopy (ICP-OES) analysis

Pulverized dental specimen (0.2 g) (Primary teeth: 49; Permanent teeth: 49) was taken with the help of precision scales, and placed in plastic tubes (Total: 98 tubes). Firstly 3 ml of H2O2 (hydrogen peroxide), and then 2 ml of HNO3 (nitrite oxide) were added to the each tube. After that, the solution was subjected to dissolution. The samples were prepared to be ready for reading by adding distilled water until the total volume was 25 ml. The solution samples prepared for the analysis were read at different wavelengths for each element (Sodium (Na), potassium (K), magnesium (Mg), calcium (Ca), and phosphate (P)) in ICP-OES device (Perkin-Elmer, ICP/OES Optima 8300). The data were recorded in ppm.

### Microhardness analysis

Vickers hardness measurements (Shimadzu HMV-G, Kyoto, Japan) were performed in a stereomicroscope. Seven samples were examined for each group. Prior to the examination, the samples were sanded with 600 and 1200 grit sandpaper. The Vickers hardness probe in the form of a diamond pyramid in the microhardness tester was applied to the tooth under a load of 25 g for 10 s in enamel and 10 g for 15 s in dentin [12, 13, 19].

A Vickers measurement was performed from three different points of the enamel and dentin (Fig. 1): Surface enamel (50 μm inner part of the enamel external surface), middle enamel (middle of the enamel), deep enamel (50 μm away from the DEJ), surface dentin (50 μm away from the DEJ), middle dentin (middle of the dentin), deep dentin (50 μm away from the pulp chamber). The average of all three Vickers hardness values obtained from the enamel and dentin was recorded as the overall hardness value of enamel and dentin.

### SEM analysis

The experimental teeth, irradiated with 30 and 60Gy, and control teeth, totally 6 teeth (3 primary and 3 permanent teeth) were fixed on the stubs using double-sided adhesive carbon disc (Agar Scientific). Subsequently, the specimens were dried in a vacuum of 10^− 2^ mbar provided by a Sputter Coater (Bal-Tec, SCD 050; Liechsteinstein). A 45 mA sputtering current was applied for 30 s. to obtain a 15 nm gold-palladium layer on the upper surface of the specimens in this equipment. The samples were examined in a Scanning Electron Microscope (SEM, LEO-Evo 40; Cambridge, United Kingdom) at magnifications of × 100, × 500 and × 1000 operating at an accelerating voltage of a 20 kV under high vacuum (10^− 5^ mbar). A secondary electron detector was employed to observe the micro-morphological characteristics of the specimens.

### Statistical analysis

Data analysis was performed using the statistical package IBM SPSS Statistics 21 (SPSS Inc., Chicago Illinois, USA). The results were expressed as means ± standard deviations. The data were firstly analyzed for the normal distribution with Shapiro**-**Wilk test. One-way ANOVA with post-hoc Tukey test was used for comparison among the groups. *P* < 0.05 values were considered as significant.

## Results

### ICP-OES result

The mean ± SD values of the elements and Ca/P weight ratio in ppm are presented in Table 1. There were statistically significant differences among the groups according to the elemental analysis. For the primary and permanent teeth, the mean of Na, K, Mg, P, and Ca elements, and Ca/P weight ratio at the end of the 6-week radiotherapy were significantly lower than what was observed in nonirradiated teeth (*p* < 0.05) (Table 1). Only in the mean Ca/P weight ratio was detected the statistically significant increase after 60Gy in permanent teeth compared to the nonirradiated teeth (Table 1).

**Table 1:** The Mean ± SD (ppm) of the elements in primary and permanent teeth

Element	Group	Primary teeth		Permanent teeth	
		Mean ± SD	**p-value	Mean ± SD	**p-value
Na	Control	4760.24 ± 149.54	< 0.001	7613.43 ± 304.77	< 0.001
	10Gy	3358.84 ± 601.87*		6965.26 ± 814.86	
	20Gy	4677.00 ± 581.66		7085.43 ± 731.39	
	30Gy	2508.84 ± 562.43*		2089.43 ± 321.02*	
	40Gy	5214.71 ± 525.79		7062.29 ± 551.12	
	50Gy	2551.24 ± 687.69*		625.79 ± 73.88*	
	60Gy	2543.30 ± 442.70*		215.00 ± 32.00*	
K	Control	148.48 ± 22.97 02	< 0.001	188.29 ± 17.62	< 0.001
	10Gy	147.93 ± 25.79		161.94 ± 23.87	
	20Gy	120.20 ± 24.60		180.19 ± 21.37	
	30Gy	102.23 ± 8.22*		160.64 ± 18.80	
	40Gy	81.33 ± 10.87*		160.83 ± 19.20	
	50Gy	104.76 ± 22.94*		147.16 ± 28.03*	
	60Gy	106.51 ± 12.77*		79.06 ± 13.28*	
Mg	Control	4367.71 ± 108.93	< 0.001	4626.43 ± 372.14	< 0.001
	10Gy	4026.89 ± 458.72		3334.86 ± 227.89*	
	20Gy	4024.85 ± 218.78		3341.29 ± 399.28*	
	30Gy	2045.25 ± 283.57*		3356.71 ± 136.57*	
	40Gy	4499.43 ± 345.28		4856.14 ± 704.16	
	50Gy	4113.91 ± 549.14		3828.71 ± 489.43*	
	60Gy	3709.77 ± 515.20*		3726.86 ± 340.46*	
P	Control	86,602.86 ± 2259.88	< 0.001	156,850.14 ± 4598.47	< 0.001
	10Gy	86,368.57 ± 8044.51		161,571.43 ± 7246.08	
	20Gy	79,994.29 ± 2483.12		158,100.00 ± 6943.82	
	30Gy	79,881.43 ± 4634.40		153,442.86 ± 7654.16	
	40Gy	80,130.00 ± 5539.45		155,257.14 ± 10,548.75	
	50Gy	73,243.86 ± 4768.31*		149,042.86 ± 5046.40	
	60Gy	82,762.00 ± 4982.26		137,357.14 ± 3353.04*	
Ca	Control	304,400.00 ± 1597.92	< 0.001	358,938.57 ± 6167.83	0.003
	10Gy	288,385.71 ± 20,510.76		345,360.00 ± 18,179.58	
	20Gy	289,742.86 ± 8233.24		346,442.86 ± 12,877.87	
	30Gy	273,314.29 ± 21,881.15*		342,138.57 ± 13,571.27	
	40Gy	256,442.86 ± 18,057.30*		349,285.71 ± 17,032.56	
	50Gy	251,714.29 ± 12,113.41*		341,071.43 ± 9620.24	
	60Gy	258,114.29 ± 18,460.90*		326,487.14 ± 10,067.46*	
Ca/P	Control	3.52 ± 0.08	< 0.001	2.29 ± 0.04	< 0.001
	10Gy	3.35 ± 0.10		2.14 ± 0.04*	
	20Gy	3.62 ± 0.03		2.19 ± 0.04*	
	30Gy	3.42 ± 0.11		2.23 ± 0.04	
	40Gy	3.20 ± 0.13*		2.25 ± 0.05	
	50Gy	3.44 ± 0.15		2.29 ± 0.03	
	60Gy	3.11 ± 0.14*		2.38 ± 0.04*	

**One-Way ANOVA

* The statistically significant difference when compared with control group

**Fig. 2:**
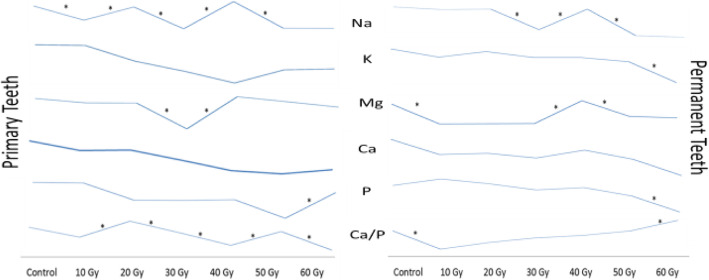
Line chart of the elements. * The statistically significant difference

All the element content of the primary and permanent teeth, except for the Ca element, showed a statistically significant decrease/increase in some radiation doses shown in Fig. 2 compared to the previous radiation dose in both primary and permanent teeth. However, a significant reduction in the Ca content of the primary teeth was noted in the radiation dose after 20Gy when compared with the nonirradiated teeth (Table 1).

### Vickers result

The mean ± SD values of the surface/middle/deep enamel, surface/middle/deep dentin, overall enamel and dentin in primary and permanent teeth are presented in Table 2.

**Table 2:** Microhardness values (mean ± SD) of the enamel and dentin of primary teeth

	Enamel				Dentin			
	Surface	Middle	Deep	Overall	Surface	Middle	Deep	Overall
**Primary Teeth**								
Control	416.71 ± 20.52	376.71 ± 19.16	343.29 ± 18.73	378.91 ± 19.19	63.31 ± 8.50	80.46 ± 9.40	71.70 ± 10.99	71.82 ± 9.59
10Gy	397.14 ± 23.79	343.43 ± 24.78*	303.43 ± 28.51	348.00 ± 25.27	56.24 ± 5.39	74.83 ± 11.55	70.16 ± 6.90	67.08 ± 7.85
20Gy	411.00 ± 21.35	355.71 ± 22.38	336.71 ± 19.75	367.81 ± 20.72	63.99 ± 7.67	71.76 ± 12.21	80.43 ± 5.03	72.06 ± 8.180
30Gy	399.43 ± 28.43	362.71 ± 14.60	312.00 ± 31.60	358.05 ± 24.46	57.14 ± 5.35	67.49 ± 7.01	65.64 ± 9.11	63.42 ± 7.03
40Gy	429.14 ± 15.28	388.71 ± 18.94	340.43 ± 33.12	386.10 ± 21.74	67.80 ± 11.26	81.31 ± 11.17	77.36 ± 9.23	75.49 ± 10.33
50Gy	426.29 ± 31.72	376.29 ± 19.57	354.29 ± 17.08	385.62 ± 22.56	62.96 ± 7.91	74.50 ± 9.56	69.84 ± 11.19	69.10 ± 9.50
60Gy	411.14 ± 24.96	367.71 ± 9.83	343.57 ± 11.33	374.14 ± 15.16	58.13 ± 4.49	78.46 ± 5.47	66.07 ± 10.97	67.76 ± 6.83
**p-value	0.128	**0.002**	**0.002**	**0.014**	0.058	0.122	**0.040**	**0.200**
**Permanent Teeth**								
Control	342.70 ± 30.20	294.63 ± 37.57	235.61 ± 15.47	290.98 ± 27.63	34.26 ± 7.36	48.31 ± 5.22	39.17 ± 7.27	40.58 ± 6.58
10Gy	295.40 ± 28.41	296.04 ± 28.88	265.60 ± 22.50	285.68 ± 25.95	36.36 ± 7.65	49.36 ± 8.96	45.17 ± 8.93	43.63 ± 8.41
20Gy	290.00 ± 34.55*	269.93 ± 37.75	232.14 ± 30.70	264.02 ± 33.86	30.94 ± 9.89	49.04 ± 10.03	40.00 ± 6.63	40.00 ± 8.64
30Gy	393.77 ± 34.85	324.68 ± 35.90	265.03 ± 29.38	327.83 ± 31.58	25.07 ± 1.03	38.51 ± 8.64	34.44 ± 9.91	32.68 ± 6.21
40Gy	399.97 ± 34.01*	366.11 ± 37.41*	302.17 ± 28.80*	356.09 ± 32.29*	33.24 ± 7.09	37.64 ± 8.61	36.64 ± 8.98	35.84 ± 8.13
50Gy	304.81 ± 25.49	237.79 ± 22.11*	233.68 ± 36.33	258.76 ± 26.39	35.47 ± 8.08	56.71 ± 4.81	48.19 ± 6.40	46.79 ± 6.31
60Gy	268.33 ± 30.46*	226.43 ± 7.15*	173.20 ± 17.68*	222.65 ± 17.76*	27.71 ± 6.52	46.14 ± 10.82	35.64 ± 6.68	36.50 ± 7.84
**p-value	**< 0.001**	**< 0.001**	**< 0.001**	**< 0.001**	**0.050**	**0.002**	**0.019**	**0.018**

**One-Way ANOVA

* The statistically significant difference when compared with control group

There were statistically significant differences in the microhardness of middle and deep enamel, and deep dentin in the primary teeth, surface/middle/deep enamel and dentin in the permanent teeth among all the groups.

There were statistically significant differences in the primary teeth overall enamel and the permanent teeth overall enamel and dentin among all the groups.

In the primary teeth enamel, the mean microhardness of the middle enamel exhibited a statistically significant decrease after 10Gy radiation compared to the nonirradiated teeth.

In the surface/middle/deep enamel and overall enamel of the permanent teeth, although significant reductions were noted in 40Gy, significant increases were seen in 60Gy, when compared with the nonirradiated teeth.

**Fig. 3:**
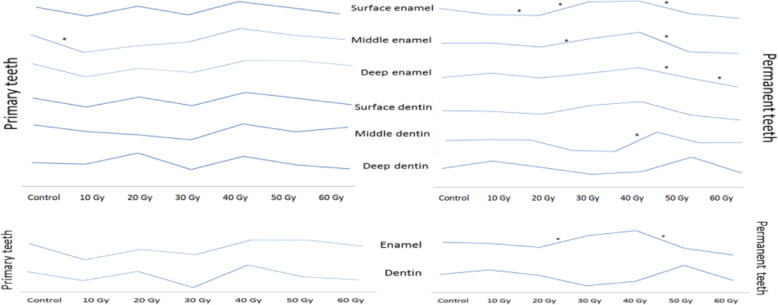
Line chart of the microhardness values. * The statistically significant difference

The mean microhardness of the permanent teeth enamel exhibited statistically significant increases/decreases after the some radiation doses (20, 30, 50, 60Gy) compared to the previous radiation dose (Fig. 3). The mean microhardness of the middle dentin in the permanent teeth showed statistically significant increases after 40Gy compared to the previous radiation dose (Fig. 3).

In the overall permanent teeth enamel, there were a statistically significant increase after 30Gy and a statistically significant decrease after 50Gy compared to the previous radiation dose.

### SEM result

**Fig. 4:**
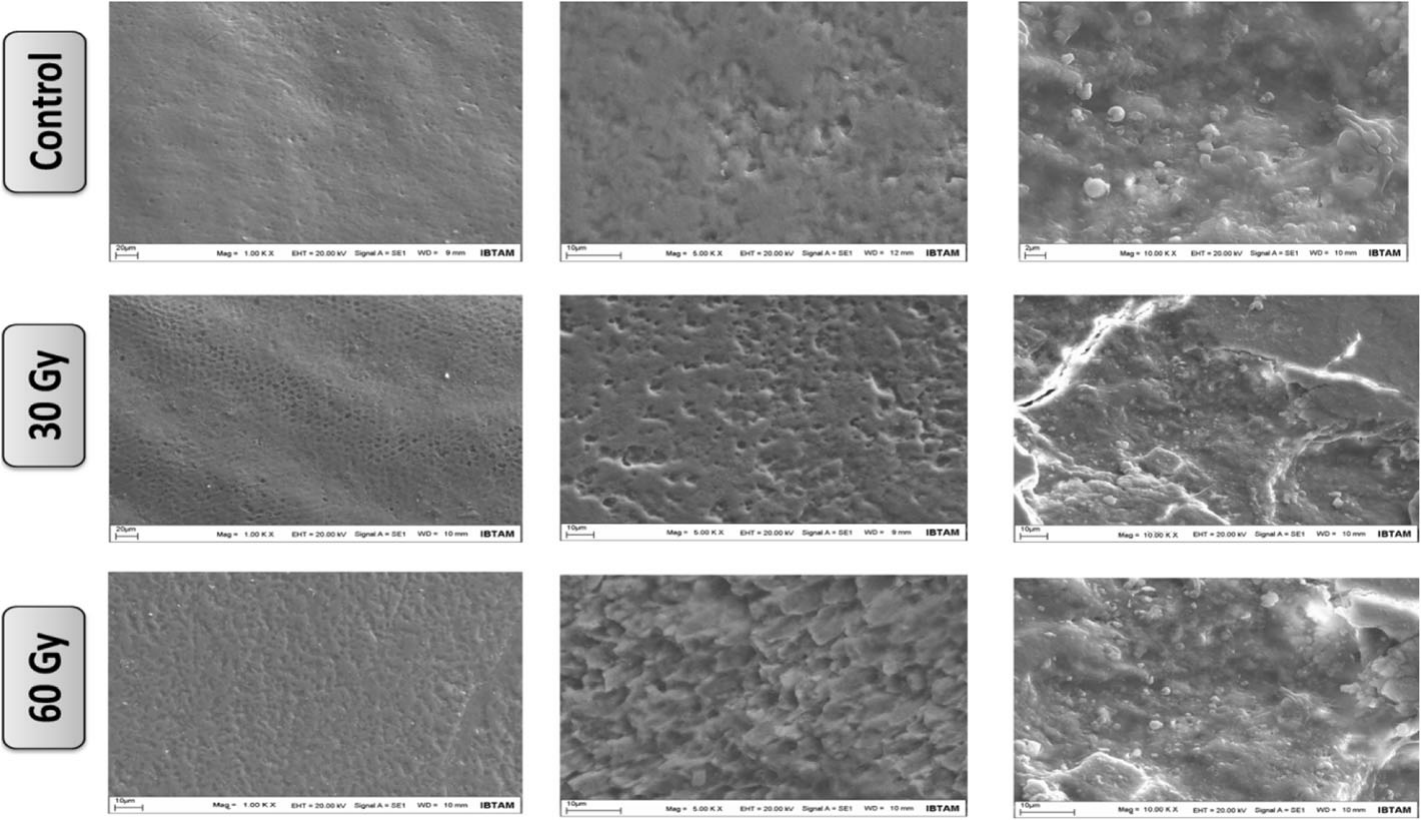
SEM images in control and 30Gy and 60Gy radiation doses at 100, 500, and 1000 magnifications

As the radiation dose increased, amorphous structures were observed on the enamel and dentin surfaces in SEM images (Fig. 4). Surface cracks were visualized on the irradiated enamel surface.

## Discussion

In this study, the changes that might be caused by radiation up to 60Gy in the extracted human primary and permanent teeth were investigated. To date, no consensus has been reached on this issue in the literature yet.

It is known that storage solutions are effective on the hardness of extracted teeth. In previous studies, extracted teeth were stored in Hanks’ balanced salt solution, PBS (pH:7.4), NaCl (0.9%), normal saline, thymol (0.2%), artificial saliva, and distilled water. A 47% decrease was reported in the dentin hardness of teeth stored in the NaCl for 30 days [20]. However, no significant change was determined in the hardness of the teeth stored in Hanks’ balanced salt solution [21]. Dry environment is also known to adversely affect the mechanical properties of dental specimens due to dehydration [22].

In order to simulate the xerostomia/ hyposalivation caused by radiotherapy in patients, Reed et al. [14] prefered to use a solution containing a small amount of storage medium instead of immersing dental specimens in PBS completely.

Marangoni-Lopes et al. [23] stated that the Ca and P concentrations significantly increased in the artificial saliva in which the specimens were kept during the enamel and dentin irradiation because of the Ca and P loss from the enamel surfaces. Thus, this solution was used to keep the specimens only during radiotherapy.

In this study, the dental samples were stored in the distilled water. During radiotherapy procedures, the teeth were wrapped in a gauze patch impregnated with artificial saliva and placed in the center of a styrofoam container filled with rice. Rice was preferred for the homogeneity of radiation dose distribution in all regions [24]. Rice grains were partially crushed to minimize the gaps between the grains. Furthermore, after each daily radiotherapy application, the teeth were put into artificial saliva and kept in the etuve at 37 °C in order to simulate the real oral environment.

It is reported that there will be little or no enamel detachment from the tooth when microhardness is measured with Vickers test rods placed perpendicularly to the cut tooth surface [13], and this affects the microhardness measurement. In our study, the measurement was performed with Vickers measurement rods which were perpendicular to the enamel cut surface.

The measurement points selected for the microhardness analysis were shaped in the light of previous studies [4, 10]. Because of the increasing amount of organic structure in the DEJ region, this region was stated to be affected more by radiotherapy than the other regions [4, 10, 12, 14]. It was reported that the enamel and dentin hardness values increased depending on the point of the measurement, and the hardness value when moved away from the DEJ region [25]. In our study, the measurements were made from the enamel and dentin regions 50 μm away from the DEJ region.

Lu et al. [12] and Gonçalves et al. [4] determined a decrease in the enamel microhardness close to the DEJ region, while de Siqueria et al. [10] determined first a decrease and then an increase. As for the dentin, while Gonçalves et al. [4] found a decrease in the microhardness close to the DEJ region after radiation, de Siqueria et al. [10] first found a decrease and then an increase. In this study, a statistically significant difference was not determined in both enamel and dentin close to the DEJ region in the primary teeth when compared with the nonirradiated teeth. However, the microhardness of the deep enamel in the permanent teeth decreased significantly after 50Gy as compared with 40Gy and decreased significantly after 60Gy as compared with 50Gy.

In the literature, some studies have reported no changes [26, 27], some have reported increases [4, 10], and some have reported decreases [12, 13, 16, 17, 23, 28–30] in the overall enamel microhardness after radiotherapy. In this study, we found that in the permanent teeth, the microhardness in the surface/middle/deep enamel and the overall enamel decreased or increased significantly after the 20, 30, 50, and 60Gy doses as compared with the previous lower doses (Fig. 3).

There were statistically significant differences in the microhardness of the middle/deep/overall enamel among the groups in the primary teeth, while there were statistically significant differences in the microhardness of the surface/middle/deep enamel and the overall enamel among the groups in the permanent teeth.

There are studies in the literature indicating a decrease in the overall dentin microhardness [4, 10, 15, 23, 31–34]. It was explained that the reason of this decrease could be the high water content of dentin (10%), decreased vascularization, obliteration of dentinal tubules due to the slowing process of irradiated odontoblast cell metabolism, and the degeneration of collagen fibers due to the effect of free radicals released after irradiation [11]. In this study, it was found that, in the permanent teeth, although there was a statistically significant difference among the groups in terms of the microhardness of the surface/middle/deep dentin and the overall dentin, there were no statistically significant differences in them after all the radiation doses when compared with the nonirradiated teeth. The microhardness in the middle dentin of the permanent teeth only increased significantly after 40Gy as compared with the increase after 30Gy. No statistical difference was encountered in the dentin microhardness of the primary teeth after radiotheraph. In addition, there was a statistically significant difference only in the microhardness of the deep dentine among the groups in the primary teeth. For this study, the permanent teeth were collected as a result of the surgical removal of the impacted third molars that had not been erupted in the mouth yet. We think that they may have been more affected by the radiation since the post-eruptive calcification or maturation of the enamel did not occur.

In the literature, there are studies examining the changes in the chemical structure of the teeth after radiation.

Velo et al. [15] examined Ca, P, O, C, Mg, and Ca/P weight ratio in the irradiated root dentin by EDX. They reported decreases in O, C, Mg elements, and Ca/P weight ratio after radiotherapy. Cambi et al. [11] examined phosphate, carbonate, and amide ratios in the dentin by Raman spectroscopy and reported that they decreased in the irradiated dentin. Reed et al. [14] determined a decrease in the protein/mineral ratio and in the carbonate/phosphate ratio in the enamel region close to the DEJ when analyzed with Raman spectroscopy in the human teeth. They attributed the decrease in the protein/mineral ratio to the structural change of collagen in both enamel and dentin. Marangoni-Lopes et al. [23] stated that radiotherapy caused a reduction in the mineral and organic contents of the enamel, and a growing increase followed by a reduction after the 0.03Gy dose in the organic contents of the dentin.

On the other hand, Lu et al. [12] reported a slight increase in the protein/mineral ratio in the enamel and a decrease in the dentin when analyzed with Raman spectroscopy. They also examined the Ca/P ratio by an Electron Probe Micro-Analyzer and reported that Ca and P elements decreased and the Ca/P ratio increased. de Barros da Cunha et al. [30] stated that radiation did not interfere with the enamel Ca and P content.

In this study, Na, K, Mg, P, and Ca elements and the Ca/P weight ratio in the analysis of the primary and permanent teeth’s hard tissues were performed with ICP-OES. It was observed that there were statistically significant differences in all the elements investigated and the Ca/P weight ratio among the groups in both primary and permanent teeth. Irregular increases and decreases in Na and Mg elements and Ca/P weight ratio in both primary and permanent teeth were observed with every 10Gy radiation dose increased. However, at the end of the 6-week radiotherapy, the five elements of the primary and permanent teeth decreased when compared with the nonirradiated teeth.

In ICP-OES, elemental analyses of all the hard tissues were performed without distinguishing between enamel and dentin. The reason for the decrease in these elements after radiotherapy can be explained by the fact that they may be replaced by heavy metals or free radicals released. Free radicals are produced by the effect of ionizing radiation. As a result of this, oxidative stress can cause structural and functional modifications by damaging important biomolecules such as DNA, proteins and lipids. Oxidative stress caused by reactive oxygen species has been reported to be effective in the etiology of heavy metal toxicities [35, 36]. Heavy metals, which are important inducers of oxidative stress, are activated to act as catalysts.

Miculescu et al. [37] stated that heavy elements accumulate faster than the major elements of teeth which is lost with aging. It can be thought that radiation may have revealed a similar effect of aging.

Previous studies have also reported that radiation doses have a greater effect on teeth as the doses are increased [4, 8, 10, 12, 15, 38]. In our study, the significant changes in elements started generally after 30Gy and these changes were observed after 40, 50, and 60Gy, too. In the microhardness assessment, the significant changes were observed only in the permanent teeth enamel after 40 and 60Gy.

Even though significant changes were observed in the inorganic structure of the teeth according to the results of the elemental analysis, these changes were slight in microhardness analyses. We estimate that this may be due to the fact that hyposalivation was not fully reflected in vitro conditions. Because the teeth were soaked in the distilled water, collagen fibers could have absorbed the water. So, the flexibility of the teeth may have increased.

This is the first study in which primary and permanent teeth are examined together. This gave us the opportunity to compare the responses of the primary and permanent teeth with the different doses of the radiation within the same study protocol. Furthermore, there is no study in the literature examining the five most significant major elements of teeth, which makes this study original. Moreover, very different findings in the previous studies indicate that there is still no clear data on the subject in the literature, and that similar studies are needed for the future.

In this study, we investigated the direct effect of the radiation on the dental hard tissue regardless of the best known side effect of radiation, which is hyposalivation. The obtained results confirmed the negative effect of the radiation on teeth. Thus, the null hypothesis was rejected since there were differences between the nonirradiated and irradiated teeth.

It is necessary to develop strategies to minimize the damage caused by radiation in the dental hard tissue for the patient’s dental health. It should be remembered that patients undergoing radiotherapy are individuals at high risk of caries, and protective applications (such as oral hygiene education, application of caries prevention agents, and non-cariogenic diet recommendations) should be focused in these patients.

The limitations of this study can be listed as follows: (i) in vivo conditions are not fully met, (ii) only the 5 major elements of the tooth were examined, but heavy metals that are thought to increase in the teeth were not examined. On the other hand, the strength of the study is that the effect of radiation doses (from 10 to 60Gy) on the morphogical, mechanical and chemical properties of both primary and permanent teeth were examined in the same study protocol, which has never been studied before.

## Conclusions

Radiotherapy has caused adverse effects on the element contents of both primary and permanent teeth tissues and on the microhardness of enamel of immature permanent teeth. The development of strategies that will minimize these negative effects of radiotherapy will be an important step for patients’ oral health. There is still no consensus on the side effects of radiation in the literature, and further studies are needed with a larger number of samples supported by in vivo studies.

## Data Availability

The raw data are available in the authors and in the Scientific Research Foundation of Inönü University. However, they are not open to the public access.

## Abbreviations

DMFT/dmft: Decayed-missing-filled teeth
MV: Megavoltage
Gy: Gray
MU/min: Monitor units / minute
ICP-OES: Inductively Coupled Plasma-Optical Emission Spectrometry
EDX: Energy dispersive X-ray
Ppm: Parts per million
Ca: Calcium
Na: Sodium
K: Potassium
Mg: Magnesium
P: Phosphate
SEM: Scanning Electron Microscope
DEJ: Dentinoenamel junction
